# Bronchoalveolar Lavage Fluid IFN-**γ** ^+^ Th17 Cells and Regulatory T Cells in Pulmonary Sarcoidosis

**DOI:** 10.1155/2014/438070

**Published:** 2014-05-05

**Authors:** Anders Tøndell, Torolf Moen, Magne Børset, Øyvind Salvesen, Anne Dorthea Rø, Malcolm Sue-Chu

**Affiliations:** ^1^Department of Thoracic Medicine, St. Olavs University Hospital, Postboks 3250 Sluppen, 7006 Trondheim, Norway; ^2^Department of Immunology and Transfusion Medicine, St. Olavs University Hospital, 7006 Trondheim, Norway; ^3^Department of Cancer Research and Molecular Medicine, Norwegian University of Science and Technology, 7489 Trondheim, Norway; ^4^Department of Laboratory Medicine, Children's and Women's Health, Norwegian University of Science and Technology, 7489 Trondheim, Norway; ^5^Department of Circulation and Imaging, Norwegian University of Science and Technology, 7489 Trondheim, Norway

## Abstract

In sarcoidosis, increased Th17 cell fractions have been reported in bronchoalveolar lavage fluid, and elevated numbers of Th17 cells producing IFN-**γ** have been observed in peripheral blood. The balance between Th1, Th17, and FoxP3^+^ CD4^+^ T cell subsets in sarcoidosis remains unclear. Bronchoalveolar lavage fluid cells, from 30 patients with sarcoidosis, 18 patients with other diffuse parenchymal lung diseases, and 15 healthy controls, were investigated with flow cytometry for intracellular expression of FoxP3. In a subset of the patients, expression of the cytokines IL17A and IFN-**γ** was investigated. The fractions of FoxP3^+^ CD4^+^ T cells and Th17 cells were both lower in sarcoidosis compared to controls (*P* = 0.017 and *P* = 0.011, resp.). The proportion of Th17 cells positive for IFN-**γ** was greater in sarcoidosis than controls (median 72.4% versus 31%, *P* = 0.0005) and increased with radiologic stage (*N* = 23, rho = 0.45, and *P* = 0.03). IFN-**γ** ^+^ Th17 cells were highly correlated with Th1 cells (*N* = 23, rho = 0.64, and *P* = 0.001), and the ratio of IFN-**γ** ^+^ Th17/FoxP3^+^ CD4^+^ T cells was prominently increased in sarcoidosis. IFN-**γ** ^+^ Th17 cells may represent a pathogenic subset of Th17 cells, yet their expression of IFN-**γ** could be a consequence of a Th1-polarized cytokine milieu. Our results indicate a possible immune cell imbalance in sarcoidosis.

## 1. Introduction

Sarcoidosis is a granulomatous disease with a predilection for the lungs and lymphatic tissue and is characterized by increased fractions and number of IFN-*γ*-producing T helper cells (Th1 cells) in inflamed tissue [1, 2]. A current hypothesis about the etiology is that an inhaled antigen, which may be persistent and poorly degradable, provokes a Th1 type immune reaction. Both mycobacterial proteins and DNA from* Propionibacterium acnes* have been identified as possible candidate antigens [3].

The discovery of the CD4^+^ T cell subsets regulatory T cells and later Th17 cells has modified the traditional concept of Th1- or Th2- polarized adaptive immune responses [4–7]. Whereas regulatory T cells, which are characterized by expression of the transcription factor FoxP3, have a pivotal role in maintaining immune homeostasis and preventing autoimmunity [8, 9]; Th17 cells produce the potent proinflammatory cytokine IL-17 and have a crucial role in host immunity towards extracellular bacterial and fungal pathogens [10]. Both Th17 cells and FoxP3^+^ CD4^+^ T cells have been implicated in various human diseases with suspected autoimmune etiology, such as rheumatoid arthritis, inflammatory bowel disease, multiple sclerosis, and psoriasis [10–12]. Intriguingly, the putative sarcoidosis antigens* Mycobacterium tuberculosis* and* Propionibacterium acnes* have both been reported to trigger strong Th17 responses [10]. Furthermore, Th17 cells recruit Th1 cells to the lungs during a mycobacterial infection and are required for proper formation of granulomas [13].

Increased Th17 cell fractions in peripheral blood and bronchoalveolar lavage fluid (BALF), and surrounding the central core of the granuloma on tissue specimens have been reported in sarcoidosis [14]. Within the Th17 cell population, there are subsets secreting different cytokines, including TNF-*α* and IFN-*γ*, with distinct effector functions [15]. IFN-*γ* ^+^ Th17 cells have been observed in elevated numbers in inflamed tissue in various conditions in man and mice [16–18]. In sarcoidosis, peripheral blood Th17 cells were reported to contain an increased fraction of IFN-*γ* ^+^ cells compared to controls, although the IFN-*γ* median fluorescent intensity of these cells was decreased [19].

Reports on regulatory T cells in sarcoidosis are conflicting. FoxP3^+^ CD4^+^ T cells are present in increased numbers in and around granulomas [20]. However, in BALF both increased [21] and decreased frequencies [22] have been reported. Interestingly, an imbalance of the regulatory T cells and the proinflammatory Th17 cells may contribute to the pathophysiology of autoimmune diseases [23–26]. These two CD4^+^ T cell subsets share common promoting factors and chemokine receptors that constitute developmental and functional links [27, 28].

In this study, we investigated the proportion of CD4^+^ T cell subsets expressing FoxP3 and, upon stimulation, IL17 or IFN-*γ* in patients with sarcoidosis, other DPLDs, and healthy control subjects. The aim of the study was to investigate the fractions of FoxP3^+^ CD4^+^ T cells, Th1, Th17, and IFN-*γ* ^+^ Th17 cells in BALF and to assess the hypothesis about an imbalance between these subsets in sarcoidosis. We also investigated if these phenotypes correlated with the radiological staging of the sarcoidosis.

## 2. Materials and Methods

### 2.1. Study Population

Patients who underwent diagnostic workup with bronchoscopy and bronchoalveolar lavage in our clinic between November 2010 and September 2012 for possible diffuse parenchymal lung disease (DPLD) were eligible for inclusion in the study if they had a BALF lymphocyte fraction greater than 5% of the leucocytes and were not receiving systemic therapy with corticosteroid or methotrexate. We included 30 patients with sarcoidosis and 18 patients with other DPLDs (hypersensitivity pneumonitis: *N* = 5; idiopathic pulmonary fibrosis: *N* = 2; nonspecific interstitial pneumonia: *N* = 1; connective tissue disease or medication-associated lung disease: *N* = 2; pneumoconiosis: *N* = 1; unspecified DPLD: *N* = 7). Patients with a concluding non-DPLD clinical diagnosis were not included. For this study, the diagnosis of sarcoidosis was considered certain if clinical presentation and thoracic imaging were consistent with pulmonary sarcoidosis and there were noncaseating granulomas in endobronchial or transbronchial biopsy specimens or from endobronchial ultrasound transbronchial aspirations of enlarged hilar or mediastinal lymph nodes [29]. Histological demonstration of granuloma was not required for patients with classic features of Löfgren's syndrome, defined as bilateral hilar lymphadenopathy with fever, erythema nodosum, and/or ankle arthritis. There were 3, 14, 9, 2, and 2 sarcoidosis patients with radiological staging 0, 1, 2, 3, and 4, respectively, according to Scadding [30], and 5 patients presented with Löfgren's syndrome.

Investigation of intracellular expression of IL-17A and IFN-*γ* after mitogen stimulation was performed in a subgroup of the patients: sarcoidosis: *N* = 23 (3 patients with Löfgren's syndrome); other DPLDs: *N* = 11 (hypersensitivity pneumonitis: *N* = 3; idiopathic pulmonary fibrosis: *N* = 1; connective tissue disease or medication associated lung disease: *N* = 2; unspecified DPLD: *N* = 5).

Eight male and 7 female healthy control subjects were recruited by advertising on the hospital website. All were nonsmokers with no history of allergy, asthma, or other lung diseases, and had normal chest radiography. All healthy control subjects and all patients except two were Caucasians of Scandinavian descent. Characteristics of the study population are displayed in Table 1.

Written informed consent was obtained from all subjects, and the study was approved by the Regional Ethics Committee (Ref.nr.: 2010/1939-4).

### 2.2. BAL Procedure

BAL was performed as previously described [31]. The choice of the lavage site was guided by the location of parenchymal pathology on high resolution computed tomography (HRCT), or if indifferent, the right middle lobe was chosen. With the bronchoscope in a wedged position in a segmental bronchus, 2-3 aliquots of 60 mL phosphate-buffered saline (hospital pharmacy at Haukeland University Hospital, Bergen, Norway) were instilled and retrieved by applying gentle suction. The second and third fractions of BALF were pooled and filtered through a Falcon Cell strainer with 100 *μ*m nylon mesh (Becton, Dickinson and Company (BD) Biosciences, Mountain view, CA) and stored at 4°C until processing within 4 hours of collection.

### 2.3. Total and Differential Cell Counts

ADVIA 120 Hematology System (Siemens AG, Erlangen, Germany) was used for determination of total cell count. Smears were prepared by cytocentrifugation (Hettich Universal 320, DJB Labcare Ltd., Buckinghamshire, England) and stained with May Grünwald/Giemsa. Differential cell counts were done on a minimum of 300 cells.

### 2.4. Mitogen Stimulation

The cells were processed according to the recommendations in the human Th17/Treg phenotyping kit (BD Biosciences) (see supplementary Table 1 in Supplementary Material available online at http://dx.doi.org/10.1155/2014/438070; tube 4). Briefly, 1-2 × 10^6^ cells were incubated at 37°C and 5% CO_2_ for 5 hours in Roswell Park Memorial Institute medium (RPMI) with 10% fetal calf serum (FCS) with and without phorbol-12-myristate-13-acetate (PMA) at 50 ng/mL and ionomycin 1 *μ*g/mL, in the presence of GolgiStop, a protein transport inhibitor (BD Biosciences). The cells were fixed by BD FoxP3 buffer set, buffer A (BD Biosciences), and stored for <6 months in a medium of 90% FCS and 10% dimethyl sulfoxide (DMSO) at −80°C until batch processing.

### 2.5. Antibody Staining and Flow Cytometry

For surface antigen staining, BALF was centrifuged for 5 min at 500 ×g, and the cell pellet incubated for 15 min with the antibodies as listed (supplementary Table 1, tubes 1-2). For detection of FoxP3^+^ CD4^+^ T cells, the unstimulated cells were incubated with permeabilization buffer (BD Biosciences) and then stained with antibodies to surface and intracellular antigens (supplementary Table 1, tube 3). In the mitogen-stimulated samples, the thawed cells were incubated with permeabilization buffer (BD Biosciences) and then stained with antibodies to surface and intracellular antigens (supplementary Table 1, tube 4). Unstimulated cells were used as negative controls for gating.

**Figure 1:**
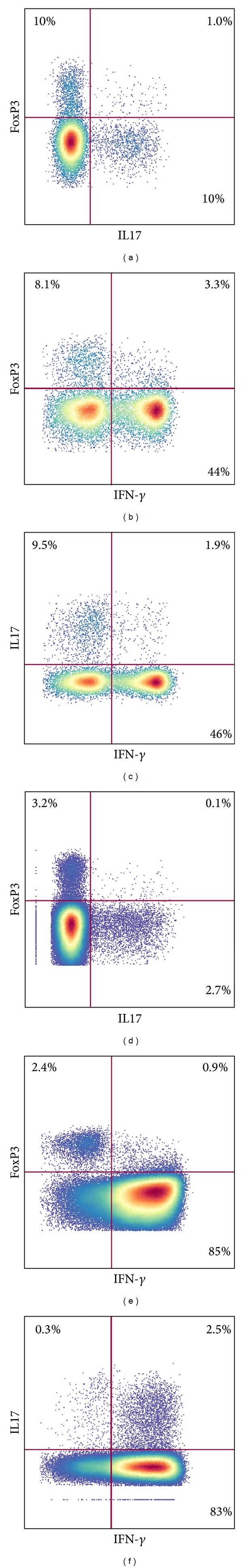
T helper cells in bronchoalveolar lavage fluid: regulatory T cells, Th1, Th17, and IFN-*γ* ^+^ Th17 cells. Flow cytometry dot plots of bronchoalveolar lavage fluid CD4^+^ T cells gated by expression of FoxP3, IL17 (synonymous to IL-17A), and IFN-*γ* from a healthy control subject ((a)–(c)) and a patient with sarcoidosis ((d)–(f)). The T cell subsets are defined as follows: Th1 cells: IFN-*γ* ^+^ CD4^+^; Th17 cells: IL17^+^ CD4^+^; regulatory T cells: FoxP3^+^ CD4^+^ and IFN-*γ* ^+^; Th17 cells: IFN-*γ* ^+^ IL-17^+^ CD4^+^. Thus, the Th17 cells include the cells in both upper quadrants of plots (c) and (f). The patient and healthy control are representative examples. Gates for IL17 and IFN-*γ* were set according to unstimulated CD4^+^ T cells. Very few cells express intracellular IL-17 or IFN-*γ* prior to stimulation (median 0.0% in fractions of CD4^+^ T cells for both IL-17 and IFN-*γ*).

A minimum of 10 000 and 50 000 cells were analysed for surface and intracellular antigens, respectively, using a FACS Canto I flow cytometer (BD Biosciences), with FACS DIVA software (BD Biosciences). Lymphocytes were identified by their low side scatter (SSc) and forward scatter (FSc), and T cells were identified as CD3^+^ lymphocytes. T cells were further gated by expression of CD4 and CD8 into the two main subsets, CD4^+^ and CD8^+^ (cytotoxic) T cells. Gating of CD4^+^ T cell subsets is displayed in Figure 1.

### 2.6. Statistical Methods

Group comparison for continuous and categorical data was done by Kruskal-Wallis test and Pearson chi-squared test, respectively. Pairwise comparison was done with Mann-Whitney* U* test if a significant difference was found with Kruskal-Wallis test. Spearman's rank correlation test was used for investigating correlation. Statistical analyses were done in R, a language and environment for statistical computing (R Core Team, R Foundation for Statistical Computing, Vienna, Austria). A *P* value of < 0.05 was considered to be statistically significant.

## 3. Results 

### 3.1. FoxP3^+^ CD4^+^ T Cells

**Figure 2:**
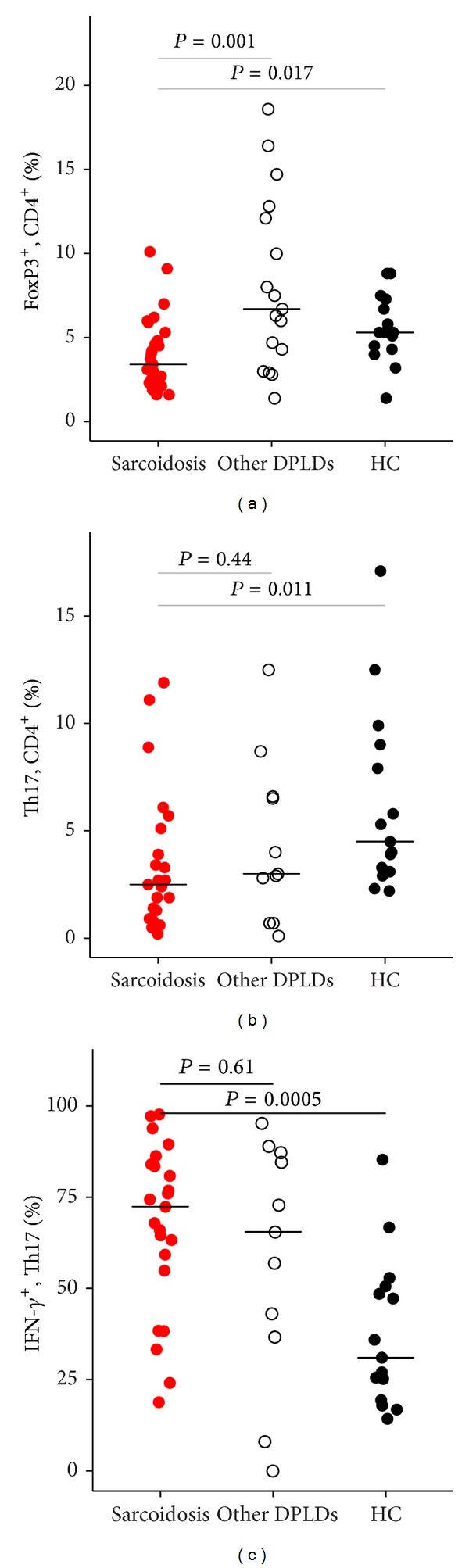
Bronchoalveolar lavage fluid fractions of FoxP3^+^ CD4^+^ T cells, Th17, and IFN-*γ* ^+^Th17 in sarcoidosis, other DPLDs, and healthy control subjects. (a) Lower fractions of regulatory T cells, defined as FoxP3^+^ CD4^+^ T cells, in sarcoidosis compared to other DPLDs and HC. (b) Lower Th17 cell fractions of CD4^+^ T cells in sarcoidosis compared to HC (median: 2.5% versus 4.5%, *P* = 0.011). (c) Higher IFN-*γ* ^+^ fraction of Th17 cells in sarcoidosis compared to HC (median: 72.4% versus 31%, *P* = 0.0005). Pairwise comparisons were done with Mann-Whitney* U* test. DPLD: Diffuse parenchymal lung disease; HC: healthy controls.

The fraction of FoxP3^+^ CD4^+^ T cells in BALF was significantly lower in patients with sarcoidosis compared to other DPLDs and HCs (median (IQR): 3.4% (2.1–4.8) versus 7.1% (4.4–12.6), *P* = 0.001 and versus 5.3% (4.4–7.0), *P* = 0.017) (Figure 2(a)).

The majority of FoxP3^+^ CD4^+^ T cells expressed CD27 and CD39 in all groups, with no significant differences between sarcoidosis and healthy controls (*P* = 0.063 and *P* = 0.51, resp.) (Table 2). Expression of CD27 and CD39 on FoxP3^+^ CD4^+^ T cells was higher in other DPLDs compared to sarcoidosis (*P* = 0.01 and *P* = 0.03, resp.), and in other DPLDs compared to healthy controls (*P* = 0.001 and *P* = 0.02, resp.).

### 3.2. Th17, IFN-*γ* ^+^ Th17, and Th1 Cells

Compared to healthy control subjects, the Th17 cell fraction of CD4^+^ T cells was 45% lower (*P* = 0.011) and the proportion of Th17 cells positive for IFN-*γ* was over twofold greater (*P* = 0.0005) in sarcoidosis (Figure 2 and Table 3). These fractions were not significantly different between patients with sarcoidosis and other DPLDs. Fractions of Th1 (IFN-*γ* ^+^ CD4^+^) cells were not significantly different between any of the groups.

Flow cytometry dot plots with a display of FoxP3^+^ CD4^+^ T cells, Th1, Th17, and IFN-*γ* ^+^ Th17 cells from a representative sarcoidosis and control subject are shown in Figure 1.

**Figure 3:**
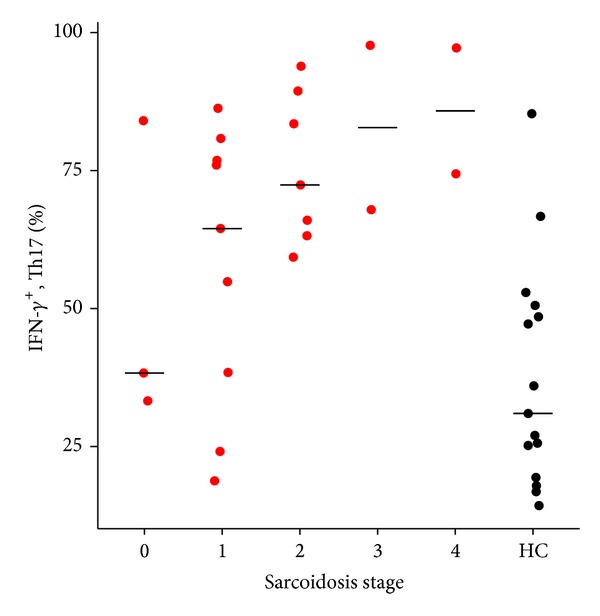
Fractions of IFN-*γ* ^+^ Th17 cells increase with radiological stage in sarcoidosis. The relationship of IFN-*γ* ^+^ Th17 cells and radiological stage in patients with sarcoidosis (*N* = 23) and healthy control subjects (*N* = 15). Fractions of IFN-*γ* ^+^ Th17 cells increase with advancing stage (*N* = 23, Spearman's rho = 0.454, and *P* = 0.03). Bars represent median. HC: Healthy control subjects.

The fraction of IFN-*γ* ^+^ Th17 cells increased with radiologic stage of sarcoidosis (*n* = 23, rho = 0.45, and *P* = 0.03) (Figure 3).

### 3.3. The Relationship between Th1, Th17, IFN-*γ* ^+^ Th17, and FoxP3^+^ CD4^+^ T Cell Subsets in Sarcoidosis

In patients with sarcoidosis, fractions of Th17 cells were moderately correlated to fractions of Th1 cells (*N* = 23, rho = 0.53, and *P* = 0.009), while we found no significant correlation between Th17 cells and FoxP3^+^ T helper cells (*N* = 23, rho = 0.25, and *P* = 0.26) or between Th1 cells and FoxP3^+^ T helper cells (*N* = 23, rho = −0.25, and *P* = 0.26).

**Figure 4:**
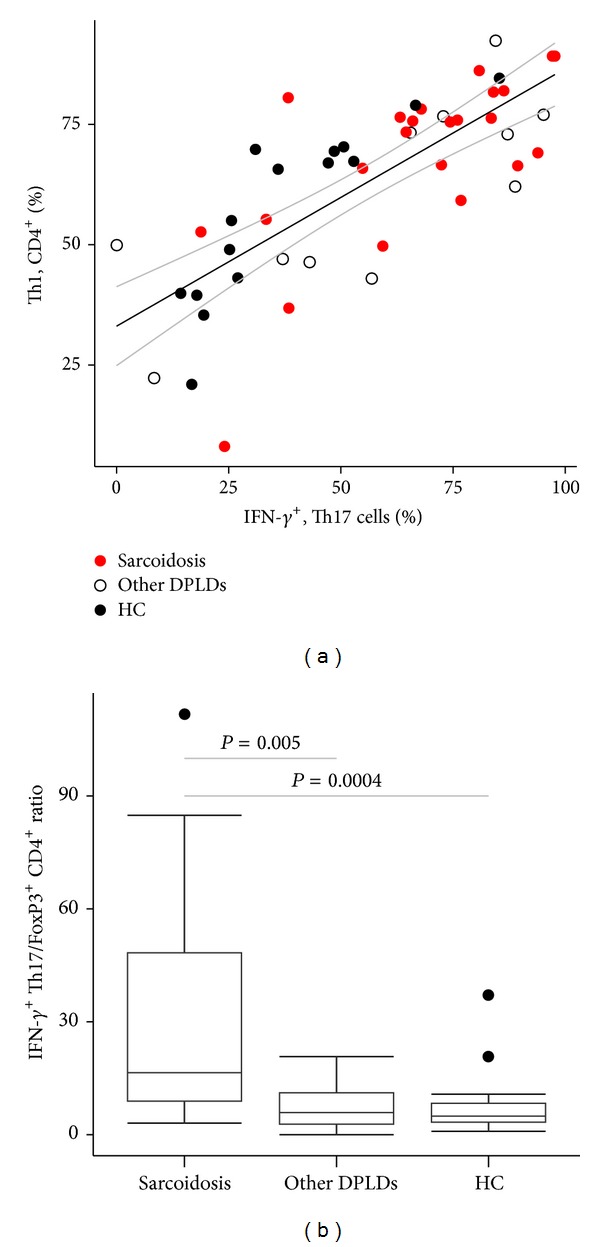
The relationship of Th1, IFN-*γ* ^+^ Th17, and FoxP3^+^ CD4^+^ T cells in bronchoalveolar lavage fluid. (a) There was a highly significant correlation between fractions of IFN-*γ* ^+^ Th17 cells and Th1 cells (IFN-*γ* ^+^ CD4^+^) (*n* = 48, Spearman's rho = 0.75, and *P* < 0.0001). Black and grey lines represent linear regression lines with 95% CI. (b) The IFN-*γ* ^+^ Th17/FoxP3^+^ CD4^+^ T cell ratio was markedly elevated in patients with sarcoidosis compared to other DPLDs or healthy control subjects. DPLD: diffuse parenchymal lung disease; HC: healthy control subjects.

IFN-*γ* ^+^ fractions of Th17 cells were highly correlated with Th1 cells in patients with sarcoidosis (*N* = 23, rho = 0.64, and *P* = 0.001). This correlation was also found in an analysis of all patients and healthy controls combined (*N* = 48, rho = 0.75, *P* < 0.0001) (Figure 4(a)), and in the other individual groups (healthy controls: *N* = 15, rho = 0.91, and *P* < 0.00001 and other DPLDs: *N* = 11, rho = 0.7, and *P* = 0.02). The ratio of Th17/FoxP3^+^ CD4^+^ T cells was not significantly different between the groups (*P* = 0.09, Kruskal-Wallis test), while the ratio of Th1/FoxP3^+^ CD4^+^ T cells was higher in sarcoidosis compared to other DPLDs and compared to HC (median (IQR): 21.7 (14.4–35.2) versus 6.2 (2.8–13.5), *P* = 0.002, and versus 9.5 (8.9–13-6), *P* = 0.009). The ratio of IFN-*γ* ^+^ Th17/FoxP3^+^ CD4^+^ T cells was considerably increased in sarcoidosis compared to other DPLDs or HC (Figure 4(b)).

The lymphocyte fraction was 2.5-fold greater in patients with sarcoidosis compared to HC (Table 4). BALF lymphocyte subset fractions in the study groups are summarized in Table 2. The CD4/CD8 ratio, HLA-DR^+^ CD4^+^, and HLA-DR^+^ CD8^+^ T cells were significantly higher in patients with sarcoidosis compared to HC (*P* < 0.0001, *P* < 0.0001, and *P* < 0.0001, resp.). Expression of HLA-DR on CD8^+^ T cells was higher in other DPLDs compared to healthy controls and sarcoidosis (*P* = 0.0001 and *P* = 0.0008, resp.) and on CD4^+^ T cells compared to healthy controls (*P* = 0.0002).

## 4. Discussion

In this study, IFN-*γ* expression in Th17 cells was over twofold greater in sarcoidosis than in healthy controls and increased with the radiologic stage of sarcoidosis. Fractions of BALF FoxP3^+^ CD4^+^ T cells and Th17 cells were lower in sarcoidosis compared to healthy controls. Furthermore, we found a high degree of correlation between fractions of IFN-*γ* ^+^ Th17 and Th1 cells, and the ratio of IFN-*γ* ^+^ Th17/FoxP3^+^ CD4^+^ T cells was prominently increased in sarcoidosis compared to other DPLDs or HCs. This may indicate a strong Th1 immune response in sarcoidosis patients resulting in deviation of CD4^+^ T cell differentiation from FoxP3^+^ CD4^+^ T cells and plain Th17 cells into IFN-*γ* ^+^ Th17 and Th1 cells.

To our knowledge, investigation of BALF IFN-*γ* ^+^ Th17 cells in sarcoidosis is limited to one study by ten Berge et al. [32]. In that study expression of IFN-*γ* was reported in a large fraction of BALF Th17 cells in 5 patients. Our finding, stating that the majority of BALF Th17 cells in sarcoidosis are IFN-*γ* ^+^, is in line with reports on Th17 cells in peripheral blood from patients with sarcoidosis [19, 32]. In the study by Richmond et al., peripheral blood IFN-*γ* ^+^ Th17 cells of sarcoidosis patients had significantly lower mean fluorescence intensity for IFN-*γ* compared to cells from control subjects, indicating a reduced capacity in these cells to produce IFN-*γ* [19]. In sarcoidosis, T cells exhibit a more differentiated phenotype in the affected organ [33, 34]. Thus, the immune process in sarcoidosis may be better characterized in studies on BALF cells.

Fractions of BALF Th17 cells were lower in sarcoidosis patients compared to controls in the present study. This is consistent with another study in which IL-17A mRNA expression in sorted CD4^+^ T cells was investigated [35], but at variance with other studies that detected intracellular expression of IL-17A by flow cytometry [14, 19, 32], several possible explanations may account for the different results. The gating of IL17^+^ from IL17^−^ CD4^+^ T cells may differ between the studies. We used unstimulated cells from the individual patients to set the gates, which we think may be an appropriate way to delineate the IL17^+^ from the IL17^−^ populations. Importantly, differences in genetic background may influence the sarcoidosis immune process. Our results are similar to those of the study from Sweden [35], but at variance with the American study [19], where approximately half of the patients had Afro-American background, and 14/37 patients had sarcoidosis of the skin. In contrast, all of our patients had pulmonary sarcoidosis. In addition, Löfgren's syndrome is more frequent in sarcoidosis in Scandinavia.

In the present study, the FoxP3^+^ fractions of CD4^+^ T cells in sarcoidosis were lower than in HC. This is in agreement with one previous study, which reported decreased frequency of FoxP3^+^ CD4^+^ T cells in BALF in patients compared to HC [22], with relative fractions resembling our results. In that study FoxP3 mRNA expression was also investigated and found to be lower in BALF CD4^+^ T cells from patients compared to HC. In another study, regulatory T cells in BALF and peripheral blood were found to be higher in patients with sarcoidosis than in control subjects [20]. However, they defined regulatory T cells as CD25^bright^ CD4^+^ T cells and confirmed the expression of FoxP3 by, on average, 86.5% of these cells by a single cell PCR approach. Later, the same group reported increased fractions of CD45RA^−^FoxP3^bright^ CD4^+^ T cells in peripheral blood and CD4^+^ FoxP3^+^ cells in lymph nodes and granulomas of patients with active sarcoidosis [36]. The definition of regulatory T cells as FoxP3^bright^ CD4^+^ T cells is also different than in the present study, where regulatory T cells are defined as FoxP3^+^ CD4^+^ T cells, making direct comparison less relevant. As documented by Darlington et al., FoxP3^+^ CD4^+^ T cells are augmented in lymph nodes compared to BALF [37], thus our finding of lower fractions of regulatory T cells in BALF in sarcoidosis may not be inconsistent with increased fractions in lymph nodes and granulomas.

IFN-*γ* ^+^ Th17 cells may represent a pathogenic subset of Th17 cells in some autoimmune diseases [17, 18]. In one study, human* Candida albicans*-specific but not* Staphylococcus aureus-specific* Th17 cells were demonstrated to produce IFN-*γ* upon stimulation [15]. During the last few years it has become increasingly clear that there exists plasticity in the differentiation of T helper cells [38]. IL-12 and IFN-*γ* influence Th17 cells to change towards IFN-*γ* ^+^ Th17 cells or Th1-like cells [16, 39]. In a study on Th17 cells recovered from diseased gut areas in colitis patients, a median of ~40% of the Th17 cells was also IFN-*γ* ^+^ [40]. In sarcoidosis, macrophages and Th1-cells are enriched in the granulomatous lung tissue, and the dominating cytokines in the feedback loop between these cells are IL-12 and IFN-*γ*. Accordingly, the reduced Th17 and increased IFN-*γ* ^+^ Th17 cells that we detected in BALF from the sarcoidosis patients might be a result of dominating Th1-driving transcription factors and cytokines. In Figure 4(a) we show that in BALF from patients and HC there seems to be a clear-cut and highly significant linear correlation between Th1 cells as a fraction of CD4^+^ T cells and IFN-*γ* ^+^ Th17 cells as a fraction of Th17 cells. This might favor an idea of a common mechanism in switching of Th17 towards IFN-*γ* ^+^ Th17 and expansion of Th1 cells. Conversely, it has recently been demonstrated in experiments with mice, in whom the Th1-driving transcription factors had been knocked out in T cells, that such naïve T cells can still develop into pathogenic IFN-*γ* ^+^ Th17 cells and that IL-23 is essential for differentiation and expansion of these cells [18]. It may thus seem to be, at least in mice, a way of differentiation for IFN-*γ* ^+^ Th17 cells independent of the IFN-*γ* driving transcription factors. The observation that the relative frequency of IFN-*γ* ^+^ Th17 cells is higher in patients with advanced sarcoidosis stage (Figure 3) might be an indication that these cells possibly have a deleterious effect in sarcoidosis or might just be the consequence of a more dominating and pathogenic Th1 immune reaction.

The ratio between IFN-*γ* ^+^ Th17 cells and FoxP3^+^ CD4^+^ T cells is markedly raised in patients with sarcoidosis in our study. Many studies have shown a reciprocal relationship between Th17 and regulatory T cells [26], and autoimmune diseases often result from an imbalance in these CD4^+^ T cell subsets [41]. An increased ratio between IFN-*γ* ^+^ Th17 cells and FoxP3^+^ CD4^+^ T cells may represent a corresponding feature of sarcoidosis. A recent study reported an increased Th17/regulatory T cell ratio in peripheral blood and BALF in sarcoidosis compared to healthy controls [42]. This was not found in our study, and fractions of Th17 cell in BALF were decreased in our study and increased in their study. Both genetic background and environmental exposures may differ between a Scandinavian and a Chinese population of patients, possibly accounting for some of these observed differences.

In the present study, CD4^+^ T cells from BALF are studied, which are more directly involved in the immunologic process of sarcoidosis than peripheral blood cells. However, there are some limitations to the study. In our clinic, the number of patients with other DPLD diagnoses is small, and patients consist of many different entities. A comparison with a sufficient number of patients with other granulomatous lung diseases would be of interest. Furthermore, the number of sarcoidosis patients in different radiological stages is small, limiting the use of our data in investigating differences between patients in different stages of the disease.

In conclusion, we found that the majority of BALF Th17 cells in sarcoidosis are IFN-*γ* ^+^, and the fraction of IFN-*γ* ^+^ Th17 cells was highly correlated with the fraction of Th1 cells. Fractions of both Th17 cells and FoxP3^+^ CD4^+^ T cells were lower in sarcoidosis than in HC, and we found a highly elevated ratio between IFN-*γ* ^+^ fractions of Th17 cells and FoxP3^+^ CD4^+^ T cells, possibly indicating an immune cell imbalance. Despite the explanation offered in this paper that the generation of IFN-*γ* ^+^ Th17 cells may be a direct consequence of a Th1-polarized cytokine milieu on Th17 cells, they may still represent a pathogenic subset of Th17 cells, and further characterization of these cells may provide a target for therapy and provide additional clues to the complex immunopathology of sarcoidosis. Functional studies on these cells and investigations of these cells in patients with different disease course are warranted.

## Supplementary Material

Supplementary material containing data on the flow cytometry antibodies used for surface and intracellular antigen staining.

## Figures and Tables

**Table 1:** Basic characteristics of the study population.

Characteristic	Sarcoidosis (*N* = 30)	Other DPLDs (*N* = 18)	Healthy controls (*N* = 15)	P*
Age, years (range)	46 (37–56)^##^	63 (48–72)^§§^	35 (30–49)	0.03

Sex (male/female)	23/7	11/7	8/7	0.25

Smokers (current/former/never)	1/9/20	4/7/7	0/3/12	0.03**

FEV1, percentage of predicted	80 (68–90)	75 (63–88)^§§^	101 (95–104)	<0.001

FEV1/FVC	0.78 (0.73–0.83)^#^	0.82 (0.78–0.88)	0.85 (0.81–0.86)	0.03

TLCO, percentage of predicted	92 (77–102)^##^	57 (53–69)^§§^	103 (98–109)	0.01

Data are shown as median (IQR) unless otherwise stated. *P values represent pairwise comparison between Sarcoidosis and healthy controls with Mann-Whitney *U* test conducted if significant differences were found with Kruskal-Wallis test. ^§§^ *P* < 0.01 compared to healthy controls. ^#^ *P* < 0.05 and ^##^ *P* < 0.01 compared to other DPLD. **P value from chi-squared test. DPLD: diffuse parenchymal lung disease; FEV1: forced expiratory volume in 1 second; FVC: forced vital capacity; TLCO: transfer factor of the lung for carbon monoxide.

**Table 2:** BALF lymphocyte subsets assessed by flow cytometry.

Characteristic	Sarcoidosis (*N* = 30)	Other DPLDs (*N* = 18)	Healthy controls (*N* = 15)	P*
CD3^+^ T cells (% of lymphocytes)	96.2 (95.3–97.5)^##^	92.3 (87.0–94.6)	92.9 (89.7–94.6)	<0.0001

CD4/CD8 ratio	6.1 (4.1–10.8)^##^	2.0 (0.5–3.3)	2.6 (2.1–3.4)	<0.0001

HLA-DR^+^ (% of CD4^+^ T cells)	75.1 (60.9–80.7)	76.1 (70.2–84.4)^§§^	44.9 (36.1–56.7)	<0.0001

HLA-DR^+^ (% of CD8^+^ T cells)	47.3 (42.2–56.3)^##^	70.0 (58.6–78.1)^§§^	30.4 (25.9–33.9)	<0.0001

FoxP3^+^ (% of CD4^+^ T cells)	3.4 (2.1–4.8)^##^	7.1 (4.4–12.6)	5.3 (4.4–7.0)	0.02

CD39^+^ (% of FoxP3^+^ CD4^+^ T cells)	61 (34–78)^#^	80 (68–84)^§^	55 (45–66)	0.51

CD27^+^ (% of FoxP3^+^ CD4^+^ T cells)	86 (80–93)^#^	95 (87–97)^§§^	78 (72–86)	0.06

Data are shown as median (IQR). *P values represent pairwise comparison between sarcoidosis and healthy controls with Mann-Whitney *U* test conducted if significant differences were found with Kruskal-Wallis test. ^§^ *P* < 0.05 and ^§§^ *P* < 0.01 compared to healthy controls. ^#^ *P* < 0.05 and ^##^ *P* < 0.01 compared to other DPLD.

**Table 3:** BALF lymphocyte subsets assessed by flow cytometry of mitogen stimulated cells.

Mitogen stimulated cells	Sarcoidosis (*N* = 23)	Other DPLDs (*N* = 11)	Healthy controls (*N* = 15)	P*
Th1 (IFN-*γ* ^+^, % of CD4^+^ T cells)	75.5 (62.6–79.4)	62.1 (46.7–75.0)	65.7 (41.5–69.6)	n.s

Th17 cells (% of CD4^+^ T cells)	2.5 (1.4–4.5)	3.0 (1.8–6.6)	4.5 (3.2–8.5)	0.01

IFN-*γ* ^+^ Th17 (% of Th17)	72.4 (57.1–83.8)	65.5 (39.9–85.9)	31.0 (22.3–49.6)	0.0005

IFN-*γ* ^+^ CD8^+^ T cells (% of CD8^+^)	83.4 (69.3–87.9)	83.1 (76.4–94.3)	85.5 (77.5–89.9)	n.s

IL17^+^ CD8^+^ T cells (% of CD8^+^)	0.8 (0.5–2.4)	0.5 (0.3–1.4)	1.1 (0.9–1.6)	n.s

Data are shown as median (IQR). *P values represent pairwise comparison between sarcoidosis and healthy controls with Mann-Whitney *U* test conducted if significant differences were found with Kruskal-Wallis test. Pairwise comparisons between sarcoidosis and other DPLD or other DPLD and healthy controls showed no significant difference. n.s: nonsignificant (Kruskal-Wallis test).

**Table 4:** BALF cellular differential counts assessed by microscopy.

	Sarcoidosis (*N* = 30)	Other DPLDs (*N* = 17)	Healthy controls (*N* = 14)	P*
Total cell concentration (10^6^/mL)	19 (15–28)	26 (11–29)	14 (12–17)	0.01

Lymphocytes (%)	35 (24–53)	34 (14–48)^§^	14 (10–25)	0.001

Neutrophils (%)	2 (1–4)^##^	6 (2–9)	3 (2–5)	0.15

Mast cells (per 10 squares)	4 (2–9)^##^	21 (8–63)^§§^	2 (0–5)	0.04

Eosinophils (%)	0 (0-1)^##^	3 (1–9)^§§^	0 (0-0)	0.14

Alveolar macrophages (%)	60 (45–73)	47 (26–74)^§§^	81 (69–85)	0.001

Data are shown as median (IQR). *P values represent pairwise comparison between sarcoidosis and healthy controls with Mann-Whitney *U* test conducted if significant differences were found with Kruskal-Wallis test. ^§^ *P* < 0.05 and ^§§^ *P* < 0.01 compared to healthy controls. ^#^ *P* < 0.05 and ^##^ *P* < 0.01 compared to other DPLD. Differential counts were missing for one patient with other diffuse parenchymal lung disease (DPLD) and one healthy control.

## References

[1] Chen ES, Moller DR (2011). Sarcoidosis—scientific progress and clinical challenges. *Nature Reviews Rheumatology*.

[2] Prince JE, Kheradmand F, Corry DB (2003). 16. Immunologic lung disease. *Journal of Allergy and Clinical Immunology*.

[3] Zissel G, Prasse A, Muller-Quernheim J (2010). Immunologic response of sarcoidosis. *Seminars in Respiratory and Critical Care Medicine*.

[4] Langrish CL, Chen Y, Blumenschein WM (2005). IL-23 drives a pathogenic T cell population that induces autoimmune inflammation. *Journal of Experimental Medicine*.

[5] Park H, Li Z, Yang XO (2005). A distinct lineage of CD4 T cells regulates tissue inflammation by producing interleukin 17. *Nature Immunology*.

[6] Harrington LE, Hatton RD, Mangan PR (2005). Interleukin 17-producing CD4^+^ effector T cells develop via a lineage distinct from the T helper type 1 and 2 lineages. *Nature Immunology*.

[7] Sakaguchi S, Miyara M, Costantino CM, Hafler DA (2010). FOXP3^+^ regulatory T cells in the human immune system. *Nature Reviews Immunology*.

[8] Miyara M, Gorochov G, Ehrenstein M, Musset L, Sakaguchi S, Amoura Z (2011). Human FoxP3^+^ regulatory T cells in systemic autoimmune diseases. *Autoimmunity Reviews*.

[9] Long SA, Buckner JH (2011). CD4^+^FOXP3^+^ T regulatory cells in human autoimmunity: more than a numbers game. *The Journal of Immunology*.

[10] Korn T, Bettelli E, Oukka M, Kuchroo VK (2009). IL-17 and Th17 cells. *Annual Review of Immunology*.

[11] Lee Y, Awasthi A, Yosef N (2012). Induction and molecular signature of pathogenic TH17 cells. *Nature Immunology*.

[12] Piccirillo CA (2008). Regulatory T cells in health and disease. *Cytokine*.

[13] Curtis MM, Way SS (2009). Interleukin-17 in host defence against bacterial, mycobacterial and fungal pathogens. *Immunology*.

[14] Facco M, Cabrelle A, Teramo A (2011). Sarcoidosis is a Th1/Th17 multisystem disorder. *Thorax*.

[15] Zielinski CE, Mele F, Aschenbrenner D (2012). Pathogen-induced human TH17 cells produce IFN-*γ* or IL-10 and are regulated by IL-1*β*. *Nature*.

[16] Bosniface K, Blumenschein WM, Brovont-Porth K (2010). Human Th17 cells comprise heterogeneous subsets including IFN-*γ*-producing cells with distinct properties from the Th1 lineage. *The Journal of Immunology*.

[17] Kebir H, Ifergan I, Alvarez JI (2009). Preferential recruitment of interferon-*γ*-expressing TH17 cells in multiple sclerosis. *Annals of Neurology*.

[18] Duhen R, Glatigny S, Arbelaez CA, Blair TC, Oukka M, Bettelli E (2013). Cutting edge: the pathogenicity of IFN-gamma-producing Th17 cells is independent of T-bet. *The Journal of Immunology*.

[19] Richmond B, Ploetze K, Isom J (2013). Sarcoidosis Th17 cells are ESAT-6 antigen specific but demonstrate reduced IFN-*γ* expression. *Journal of Clinical Immunology*.

[20] Miyara M, Amoura Z, Parizot C (2006). The immune paradox of sarcoidosis and regulatory T cells. *The Journal of Experimental Medicine*.

[21] Rappl G, Pabst S, Riemann D (2011). Regulatory T cells with reduced repressor capacities are extensively amplified in pulmonary sarcoid lesions and sustain granuloma formation. *Clinical Immunology*.

[22] Idali F, Wahlström J, Müller-Suur C, Eklund A, Grunewald J (2008). Analysis of regulatory T cell associated forkhead box P3 expression in the lungs of patients with sarcoidosis. *Clinical & Experimental Immunology*.

[23] Eisenstein EM, Williams CB (2009). The T(reg)/Th17 cell balance: a new paradigm for autoimmunity. *Pediatric Research*.

[24] Nistala K, Wedderburn LR (2009). Th17 and regulatory T cells: rebalancing pro- and anti-inflammatory forces in autoimmune arthritis. *Rheumatology*.

[25] Afzali B, Lombardi G, Lechler RI, Lord GM (2007). The role of T helper 17 (Th17) and regulatory T cells (Treg) in human organ transplantation and autoimmune disease. *Clinical and Experimental Immunology*.

[26] Schmitt V, Rink L, Uciechowski P (2013). The Th17/Treg balance is disturbed during aging. *Experimental Gerontology*.

[27] Weaver CT, Harrington LE, Mangan PR, Gavrieli M, Murphy KM (2006). Th17: an effector CD4 T cell lineage with regulatory T cell ties. *Immunity*.

[28] Weaver CT, Hatton RD (2009). Interplay between the TH17 and TReg cell lineages: a (co-)evolutionary perspective. *Nature Reviews Immunology*.

[29] (1999). Statement on sarcoidosis. *The American Journal of Respiratory and Critical Care Medicine*.

[30] Scadding JG (1961). Prognosis of intrathoracic sarcoidosis in England. A review of 136 cases after five years' observation. *British Medical Journal*.

[31] Tøndell A, Rø AD, Åsberg A, Børset M, Moen T, Sue-Chu M (2014). Activated CD8^+^ T cells and NKT cells in BAL fluid improve diagnostic accuracy in sarcoidosis. *Lung*.

[32] ten Berge B, Paats MS, Bergen IM (2012). Increased IL-17A expression in granulomas and in circulating memory T cells in sarcoidosis. *Rheumatology*.

[33] Wikén M, Grunewald J, Eklund A, Wahlström J (2012). Multiparameter phenotyping of T-cell subsets in distinct subgroups of patients with pulmonary sarcoidosis. *Journal of Internal Medicine*.

[34] Wikén M, Ostadkarampour M, Eklund A (2012). Antigen-specific multifunctional T-cells in sarcoidosis patients with Löfgren’s syndrome. *European Respiratory Journal*.

[35] Wikén M, Idali F, Al Hayja MA, Grunewald J, Eklund A, Wahlström J (2010). No evidence of altered alveolar macrophage polarization, but reduced expression of TLR2, in bronchoalveolar lavage cells in sarcoidosis. *Respiratory Research*.

[36] Taflin C, Miyara M, Nochy D (2009). FoxP3^+^ regulatory T cells suppress early stages of granuloma formation but have little impact on sarcoidosis lesions. *American Journal of Pathology*.

[37] Darlington P, Haugom-Olsen H, von Sivers K (2012). T cell phenotypes in bronchoalveolar lavage fluid, blood and lymph nodes in pulmonary sarcoidosis—indication for an airborne antigen as the triggering factor in sarcoidosis. *Journal of Internal Medicine*.

[38] O’Shea J, Paul WE (2010). Mechanisms underlying lineage commitment and plasticity of helper CD4^+^ T cells. *Science*.

[39] Lexberg MH, Taubner A, Albrecht I (2010). IFN-*γ* and IL-12 synergize to convert in vivo generated Th17 into Th1/Th17 cells. *European journal of immunology*.

[40] Annunziato F, Cosmi L, Santarlasci V (2007). Phenotypic and functional features of human Th17 cells. *Journal of Experimental Medicine*.

[41] Komatsu N, Okamoto K, Sawa S (2014). Pathogenic conversion of Foxp3^+^ T cells into TH17 cells in autoimmune arthritis. *Nature Medicine*.

[42] Huang H, Lu Z, Jiang C, Liu J, Wang Y, Xu Z (2013). Imbalance between Th17 and regulatory T-Cells in sarcoidosis. *International Journal of Molecular Sciences*.

