# Supplementary material for: Bronchoalveolar Lavage Fluid IFN-γ + Th17 Cells and Regulatory T Cells in Pulmonary Sarcoidosis
Source: Mediators Inflamm. 2014 May 5;2014:438070. doi: 10.1155/2014/438070 (PMC4027000; doi:10.1155/2014/438070)
Supplement: Supplementary file 1 — Supplementary material containing data on the flow cytometry antibodies used for surface and intracellular antigen staining. [file 438070.f1.pdf]

Supplementary Table 1: Antibody panel for surface and intracellular antigen staining

Tube	Antibody conjugates						Staining
	**FITC**	**PE**	**PerCP-Cy5.5**	**PE-Cy7**	**APC**	**APC-Cy7**	
**1**	Isotype control[1]	Isotype control[1]	Isotype control[2]	Isotype control[2]	Isotype control[1]	CD45[2]	Surface
**2**	CD8[1]	CD4[1]	HLA-DR[2]		CD3[1]		Surface
**3**	CD4[2]	FoxP3[2]	CD8[2]	CD27[2]	CD39[2]	CD25[2]	Intracellular
**4**	CD4[2]	IL-17A[2]	CD8[2]	IFN-γ[2]	FoxP3[2]	CD3[2]	Intracellular +mitogen

Footnote Supplementary Table 1: [1]Multimix, Dako Denmark AS, Glostrup Denmark, [2]BD Biosciences, San Jose, USA. FITC: Fluorescein isothiocyanate, PE: phycoerythrobilin, PerCP-Cy5.5: Peridinin chlorophyll protein with cyanine dye (Cy5.5), APC: Allophycocyanin.
